# MRI susceptibility-related artifacts following prolonged use of silver-containing dressings: A case report

**DOI:** 10.1097/MD.0000000000050595

**Published:** 2026-09-04

**Authors:** Bo Liu, Jiahao Wang, Jinning Jiang, Tianchang Wang, Jingwu Zhang, Xiufei Liu, Weicai Xue

**Affiliations:** a Department of Anorectal Surgery I, Hebei Provincial Hospital of Traditional Chinese Medicine, Shijiazhuang, Hebei, China; b Department of Anorectal Surgery, Xingtai Ninth Hospital, Xingtai, Hebei, China.

**Keywords:** case report, magnetic susceptibility artifacts, MRI artifact, pilonidal sinus surgery, silver-containing dressings

## Abstract

**Rationale:**

Silver-containing dressings are widely used in postoperative and chronic wound care because of their antimicrobial properties. Although these dressings are generally considered compatible with magnetic resonance imaging (MRI) under standard conditions, prolonged use in chronic open wounds may be associated with local retention or deposition of silver-containing material. This could contribute to susceptibility-related signal loss or distortion on MRI and interfere with the evaluation of underlying anatomical structures. However, clinical evidence supporting this association remains limited, and a definitive causal relationship has not been established.

**Patient concerns:**

A 33-year-old man presented with a nonhealing wound after pilonidal sinus excision. He had used silver-containing dressings intermittently for postoperative wound care over an 8-month period. Because of delayed wound healing and recurrent symptoms, pelvic MRI was performed to evaluate the cause.

**Diagnoses:**

MRI demonstrated extensive signal loss and distortion in the sacrococcygeal region, suggestive of susceptibility-related artifacts, which obscured anatomical structures and limited diagnostic assessment. After removal of wound-related material by debridement, follow-up MRI demonstrated a fistulous tract extending from the anal canal to the previous wound site. The patient was diagnosed with an anal fistula associated with a chronic nonhealing postoperative wound.

**Interventions:**

Given the possibility that wound-related or silver-containing dressing-associated material contributed to the MRI artifacts, wound debridement was performed. After the follow-up MRI clarified the fistulous tract, the patient underwent fistula surgery.

**Outcomes:**

Follow-up MRI after debridement showed near-complete reduction of the artifacts and allowed visualization of the fistulous tract. The patient underwent surgical treatment, and follow-up showed satisfactory wound healing.

**Lessons:**

Prolonged use of silver-containing dressings may be associated with retention or deposition of dressing-related material, which could represent a possible contributor to susceptibility-related MRI artifacts in selected patients. However, silver deposition was not directly confirmed by elemental analysis or susceptibility-sensitive MRI sequences, and alternative causes could not be completely excluded. Clinicians and radiologists should consider this potential source of imaging interference in patients with chronic wounds and a history of long-term silver dressing use. Careful wound assessment and removal of residual wound-related material before MRI may be helpful when artifact-related diagnostic interference is suspected.

## 1. Introduction

Silver-containing dressings are commonly employed in postoperative and chronic wound management because of their broad-spectrum antimicrobial activity and wound-healing properties.^[1]^ Although considered safe and effective, prolonged application may be associated with local retention or deposition of silver-containing material, particularly when the skin barrier is disrupted. Magnetic resonance imaging (MRI) plays a crucial role in the assessment of pelvic and perianal conditions, yet its images are vulnerable to susceptibility-related artifacts, which are commonly associated with metallic implants, air-tissue interfaces, or blood degradation products.^[2]^ To our knowledge, clinical reports suggesting an association between prolonged exposure to silver-containing dressings and MRI susceptibility-related artifacts are extremely limited.

This report presents a postoperative pilonidal sinus case in which marked reduction of imaging artifacts after debridement raised the possibility of an association with removable wound-related material, including possible silver-containing dressing residue or deposition.

## 2. Case report

A 33-year-old male underwent pilonidal sinus excision at another hospital. According to the available medical records and the patient’s history, a dressing described as a silver-containing dressing was repeatedly used for postoperative wound care at an outside hospital over an approximately 8-month period before the initial MRI examination. Although the exact manufacturer, commercial product name, silver formulation, total amount used, and precise application frequency could not be reliably verified because of the retrospective nature of this case and the outside-hospital treatment setting, the history of prolonged exposure to a silver-containing dressing was consistently documented. Therefore, the exposure in this case was described as prolonged intermittent use of a silver-containing dressing, while no product-specific or dose-dependent conclusion was drawn.

**Figure 1.**
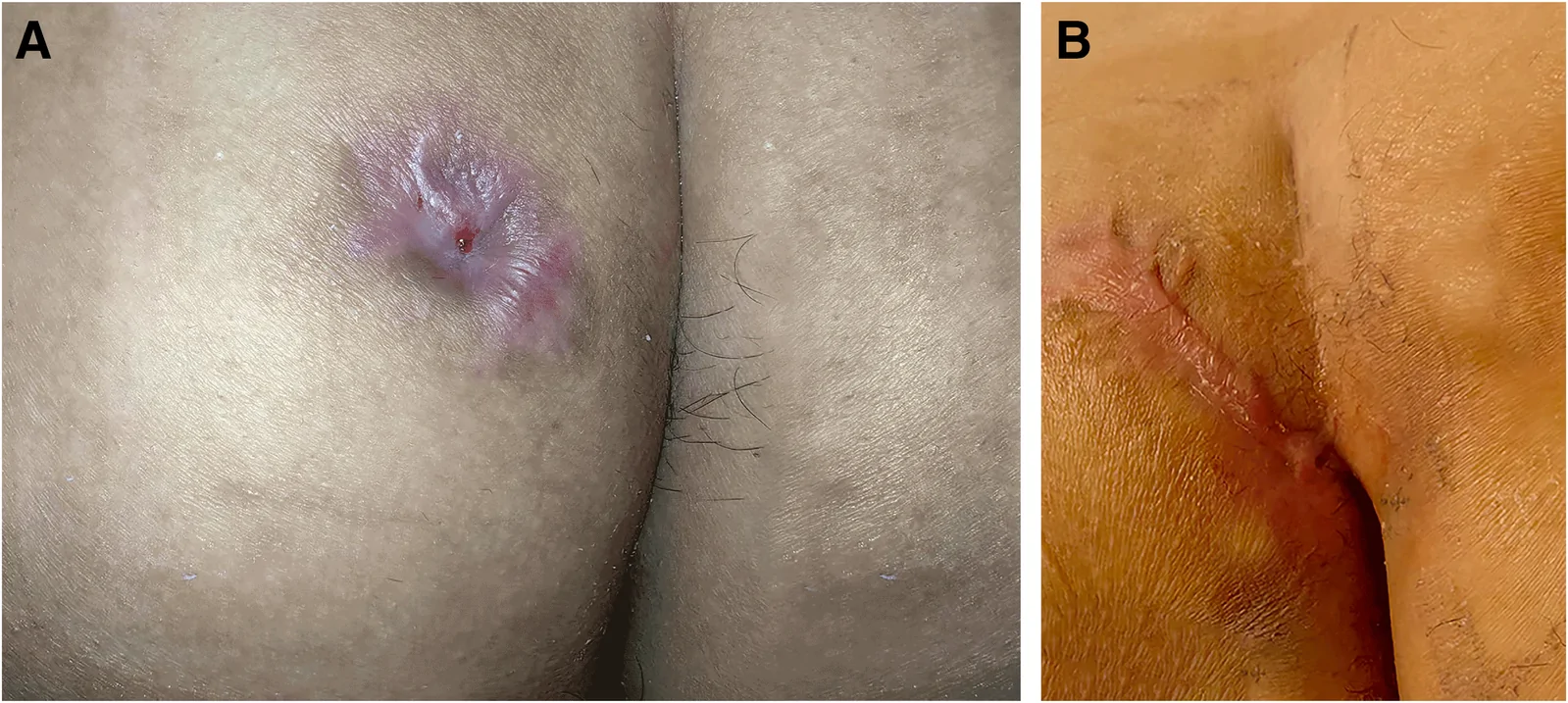
Clinical wound images. (A) Nonhealing postoperative wound at the initial visit. (B) Complete wound healing after anal fistula surgery during outpatient follow-up.

The patient later presented with a nonhealing wound characterized by recurrent ulceration and mild bloody discharge. Physical examination in the knee-chest position conducted in our hospital (Fig. 1A) revealed a small ulcer approximately 5 cm from the anal verge, slightly left of the midline, with minimal serosanguineous discharge and mild induration. No purulent discharge or malodor was detected. To evaluate the sinus tract and its relation to adjacent structures, pelvic MRI was performed.

Both pre- and post-debridement pelvic MRI examinations were performed on the same 1.5-T MRI system (GE 1.5T Signa HDxt, GE Healthcare) using a body coil. Both examinations were noncontrast pelvic MRI examinations, and the images were exported from the picture archiving and communication system. The 2 examinations used a comparable pelvic MRI protocol, although some acquisition parameters differed between the pre- and post-debridement studies. The main sequences used for diagnostic interpretation and figure presentation included oblique axial diffusion-weighted imaging (DWI) and oblique sagittal T2-weighted short tau inversion recovery (STIR) imaging. For the pre-debridement MRI, the main acquisition parameters were as follows: oblique axial DWI, repetition time/echo time (TR/TE) 3167.0/105.2 ms, slice thickness 4.0 mm, slice spacing 5.0 mm, field of view 220 mm, matrix 320 × 256, and receiver bandwidth 50 kHz; oblique sagittal T2-weighted STIR imaging, TR/TE 2590.0/81.5 ms, slice thickness 4.0 mm, slice spacing 5.0 mm, field of view 200 mm, matrix 320 × 256, and receiver bandwidth 50 kHz. For the post-debridement MRI, the corresponding parameters were as follows: oblique axial DWI, TR/TE 1502.0/90.4 ms, slice thickness 2.0 mm, slice spacing 2.0 mm, field of view 200 mm, matrix 288 × 192, and receiver bandwidth 41.67 kHz; oblique sagittal T2-weighted STIR imaging, TR/TE 2799.0/105.6 ms, slice thickness 3.5 mm, slice spacing 4.2 mm, field of view 220 mm, matrix 300 × 300, and receiver bandwidth 35.71 kHz. No susceptibility-sensitive sequences, such as T2*-weighted gradient-echo imaging, susceptibility-weighted imaging, or quantitative susceptibility mapping, were performed.

**Figure 2.**
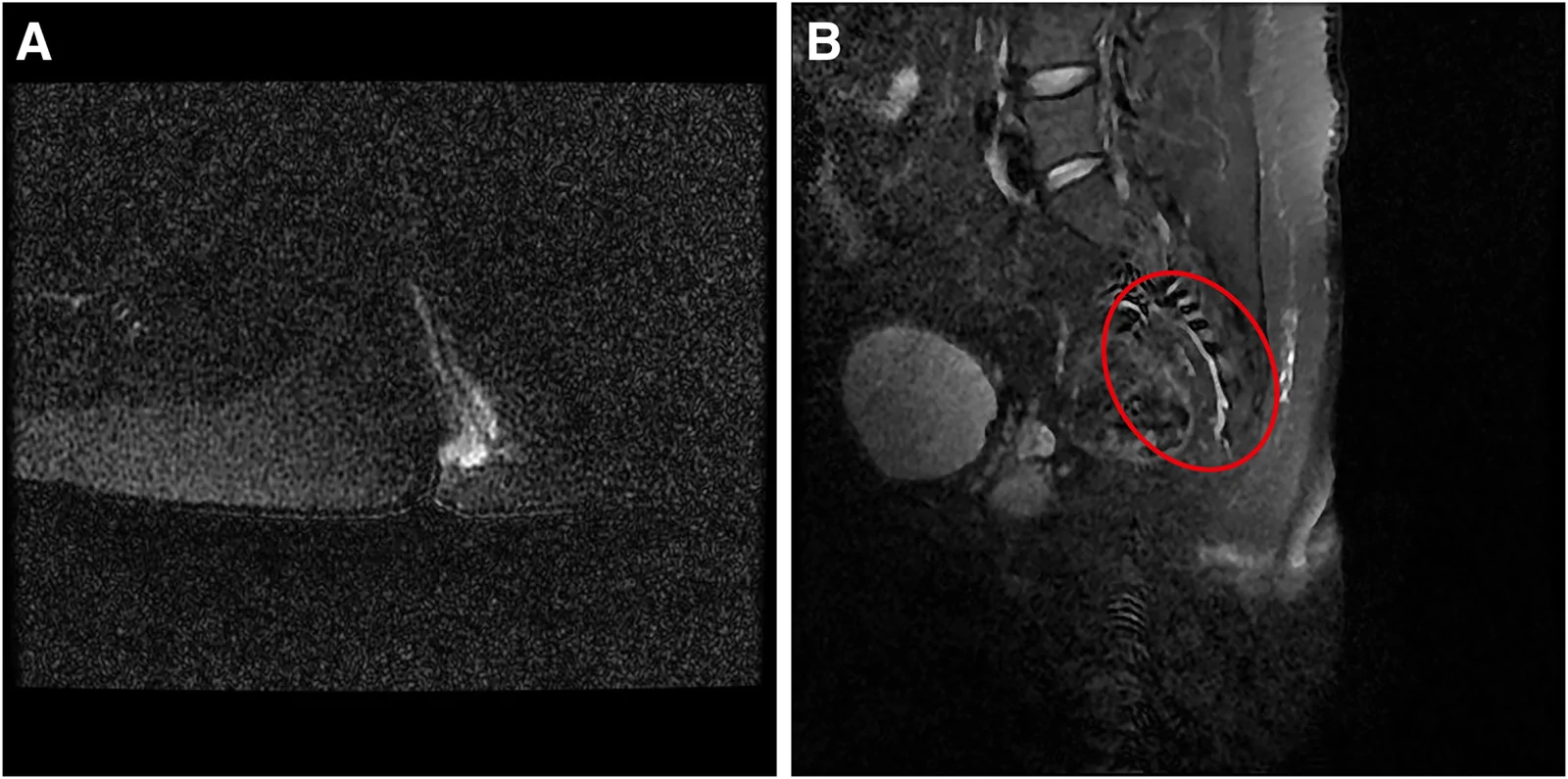
Initial pelvic MRI showing suspected susceptibility-related artifacts. (A) Oblique axial diffusion-weighted image showing diffuse signal loss in the sacrococcygeal region. (B) Oblique sagittal T2-weighted STIR image showing radial signal-loss artifacts (red oval). MRI = magnetic resonance imaging, STIR = short tau inversion recovery.

The initial MRI demonstrated a strip-like lesion beneath the coccyx, measuring approximately 5.1 × 1.1 cm, which appeared hyperintense on both T1- and T2-weighted images and extended anteriorly to the pelvic floor fascia, suggesting residual infection or recurrent pilonidal sinus. Oblique axial DWI showed diffuse signal voids (Fig. 2A), whereas oblique sagittal T2-weighted imaging demonstrated radial signal loss (Fig. 2B), rendering the local anatomy indistinct. Given the prolonged use of silver-containing dressings, deposition of silver-containing material was considered one possible explanation for the observed signal loss and distortion. However, this hypothesis could not be directly confirmed because susceptibility-sensitive sequences and elemental analyses were not performed.

**Figure 3.**
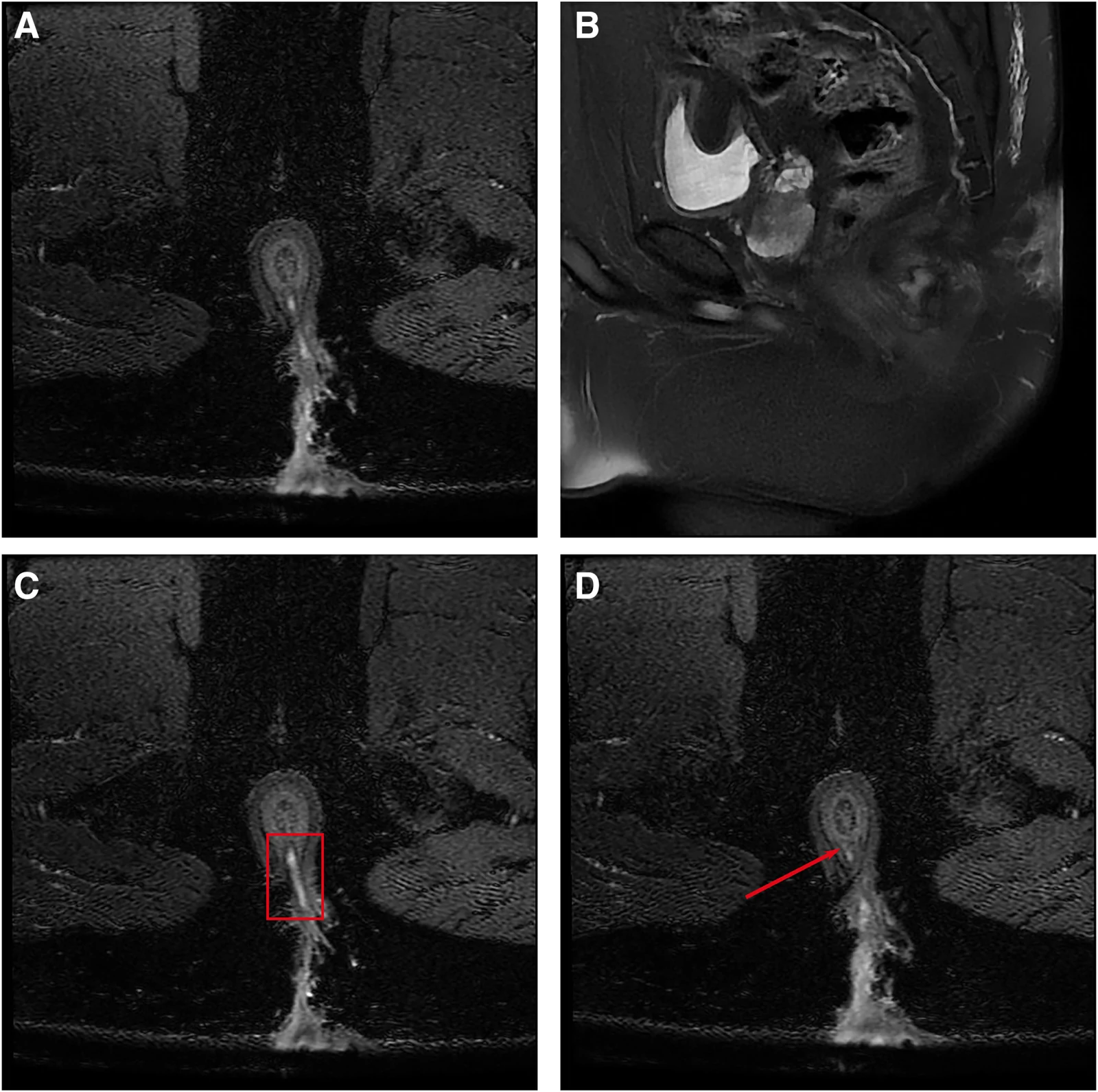
Follow-up pelvic MRI after wound debridement. Panels (A), (C), and (D) are oblique axial diffusion-weighted images, and panel (B) is an oblique sagittal T2-weighted STIR image. (A, B) Images showing near-complete resolution of the artifact. (C) Image showing the course of the fistulous tract (red rectangle). (D) Image showing the location of the internal opening of the anal fistula (red arrow). MRI = magnetic resonance imaging, STIR = short tau inversion recovery.

On the third day after wound debridement, follow-up pelvic MRI showed a marked reduction in the previously observed susceptibility-related artifacts (Fig. 3A and B). The follow-up scan demonstrated a fistulous tract extending from the left posterior anal canal (6–7 o’clock position) through the intersphincteric space to the left gluteal cleft (Fig. 3C and D). The coccyx remained unaffected. DWI revealed mildly increased signal intensity, consistent with active inflammation. The patient subsequently underwent fistulotomy, and the postoperative wound healed completely (Fig. 1B).

## 3. Discussion

Susceptibility-related MRI artifacts are commonly encountered in the presence of materials or interfaces with magnetic susceptibility differences from surrounding tissues, such as metallic implants, orthodontic materials, air-tissue interfaces, or blood degradation products.^[2]^ These differences may result in local magnetic field inhomogeneity, causing signal loss, geometric distortion, or obscuration of adjacent anatomical structures.

Previous studies have suggested that silver-containing wound dressings are generally compatible with MRI under standard conditions. For example, Bailey et al evaluated the MRI compatibility of silver-based wound dressings and reported only limited image distortion during in situ use.^[3]^ These findings indicate that routine or short-term use of silver-containing dressings does not necessarily produce clinically significant MRI artifacts.

However, the clinical context of the present case differed from the conditions described in previous compatibility studies. In many compatibility evaluations, the dressing is assessed as an intact material under controlled or short-term conditions. In contrast, the present patient had a chronic open postoperative wound and a history of repeated exposure to a silver-containing dressing over approximately 8 months. In this setting, disruption of the skin barrier, prolonged wound exudation, repeated dressing changes, and possible retention of dressing-related material within the wound may have created conditions that differed substantially from standard in situ dressing use.

The apparent difference between the present observation and previous reports may therefore be related to differences in exposure duration, wound condition, tissue contact, and the possible presence of residual wound-related material. In this case, the initial MRI showed extensive signal loss and radial distortion in the sacrococcygeal region, whereas follow-up MRI after wound debridement showed marked reduction of these artifacts. This temporal relationship suggests that removable material within or around the wound contributed to the imaging findings, rather than confirming silver deposition as the sole cause.

Several alternative explanations were considered when interpreting the observed MRI abnormality. Postoperative fluid collections, inflammatory or granulation tissue, and nonspecific postoperative changes may have contributed to part of the surrounding soft-tissue signal abnormality. However, these processes more commonly present with fluid-like or increased T2/STIR signal and therefore appear less likely, by themselves, to fully account for the predominant signal voids and radial geometric distortion observed in this case.

Air within the open wound cavity also represents an important possible contributor because the susceptibility difference between air and adjacent soft tissue may produce local field inhomogeneity, signal loss, and distortion. On the basis of the available conventional MRI sequences, an air-related effect could not be reliably distinguished from other wound-related causes or completely excluded. Hemorrhagic products may likewise produce susceptibility-related signal loss. The marked reduction of the abnormality after debridement may be less consistent with persistent tissue-bound blood products as the sole explanation; nevertheless, superficial hemorrhagic debris or blood-containing devitalized tissue could have been removed during debridement and therefore remain possible contributors.

Retained foreign material, wound debris, and residual dressing-related material should also be considered. Depending on their composition and distribution, these materials may contribute to low signal intensity, local susceptibility effects, or entrapment of small amounts of air. The substantial reduction in signal loss and distortion after debridement supports an association with removable wound-related material, although it does not establish the nature or composition of that material. Taken together, postoperative changes, air, hemorrhagic products, retained foreign material, and wound- or dressing-related debris appear less likely to provide a complete explanation individually, but none can be completely excluded. In view of the prolonged history of silver-containing dressing use, silver-containing material remains a plausible contributor rather than a confirmed or exclusive cause.

Experimental and histological studies have shown that silver can accumulate locally in damaged skin or after prolonged exposure to silver-containing products, and particulate silver deposits have been identified in tissue.^[4–6]^ On this basis, local retention or deposition of silver-containing material was considered a plausible contributor to the susceptibility-related artifacts observed in this case. Nevertheless, this interpretation remains hypothetical. The exact dressing product and silver formulation could not be verified because the dressing had been prescribed and applied at an outside hospital, which limits product-specific and dose-response interpretation. However, the history of prolonged exposure to a silver-containing dressing was documented, and the unavailable product details do not exclude the possibility that silver-containing dressing-related material contributed to the observed MRI findings.

Another important limitation is that susceptibility-sensitive MRI sequences were not performed. The attribution of the observed signal loss and distortion to susceptibility-related effects was based on conventional MRI appearances and the marked post-debridement change. T2*-weighted gradient-echo imaging, susceptibility-weighted imaging, or quantitative susceptibility mapping would have provided more direct characterization of susceptibility effects. In addition, routine hematoxylin–eosin histopathology cannot reliably determine elemental composition. Definitive confirmation of silver deposition would require elemental analyses such as scanning electron microscopy with energy-dispersive X-ray spectroscopy, inductively coupled plasma mass spectrometry, or comparable techniques.

From a clinical perspective, this case suggests that clinicians and radiologists should consider dressing-related material as a potential source of MRI interference in selected patients with chronic wounds and prolonged exposure to silver-containing dressings. Careful wound assessment and removal of residual wound-related or dressing-related material may be helpful when unexpected artifact-related diagnostic interference is suspected. In accordance with antimicrobial stewardship principles, the continued need for silver-containing dressings should be reassessed after an initial treatment period, such as after approximately 2 weeks. If wound healing stagnates or infection control has been achieved, the dressing regimen should be reviewed and adjusted as appropriate, consistent with European Wound Management Association guidance.^[7]^

Because the exact composition of the removed material remains unknown, the proposed mechanism should be regarded as hypothesis-generating. These findings should therefore be considered preliminary until further supported by susceptibility-sensitive MRI and/or elemental analysis.

## 4. Conclusion

In patients with prolonged exposure to silver-containing dressings, local retention or deposition of silver-containing material may be considered as a possible explanation for MRI susceptibility-related artifacts. Although further confirmation using susceptibility-sensitive MRI and elemental analysis is required, the present findings highlight an important diagnostic consideration. This case may increase clinical awareness of the potential association between prolonged use of silver-containing dressings and susceptibility-related MRI artifacts and highlights the need for further investigation.

## Author contributions

**Methodology:** Bo Liu, Weicai Xue.

**Software:** Tianchang Wang.

**Investigation:** Jingwu Zhang.

**Formal analysis:** Xiufei Liu.

**Writing – original draft:** Jiahao Wang, Jinning Jiang.
